# Enhanced treatment outcomes for schizophrenia through combined long-acting injectable antipsychotic medications and home visits: retrospective cohort study

**DOI:** 10.1192/bjo.2025.10809

**Published:** 2025-08-15

**Authors:** Hiroyuki Harada, Shigemasa Katayama, Tadafumi Kato

**Affiliations:** Department of Psychiatry and Behavioural Science, Juntendo University Graduate School of Medicine, Tokyo, Japan; Department of Psychiatry, Seijin Hospital, Tokyo, Japan

**Keywords:** Schizophrenia, long-acting injectable antipsychotic medication (LAI), home visit, treatment failure, retrospective cohort study

## Abstract

**Background:**

Long-acting injectable antipsychotic medications (LAIs) are more beneficial than oral medications for people with schizophrenia. However, some individuals are unable to visit out-patient clinics due to their symptoms, resulting in missed monthly LAI injections and subsequent relapse. Home visits for administration of LAIs could potentially reduce treatment failure, but there are no comparative studies on their effectiveness.

**Aims:**

This study aims to evaluate whether home visit administration of LAIs, compared with the out-patient clinic, reduces treatment failure for those with schizophrenia.

**Method:**

We conducted a retrospective cohort study using electronic medical records from Seijin Hospital. Patients diagnosed with schizophrenia and treated with LAIs during hospitalisation between 1 April 2020 and 31 March 2023 were included. Following discharge, patients were followed for 1 year, either under home visits or out-patient clinic visits. The primary outcome was defined as treatment failure, including psychiatric rehospitalisation, discontinuation of treatment or death. Multivariate Cox proportional hazards regression analysis was performed to evaluate treatment failure risks.

**Results:**

A total of 125 patients in the home visit group and 117 in the out-patient group were included. During the follow-up period, home visits significantly reduced the risk of treatment failure (hazard ratio 0.62, 95% CI 0.40–0.97). However, having two or more psychiatric hospitalisations (hazard ratio 2.32, 95% CI 1.28–4.37) and living alone following discharge (hazard ratio 1.77, 95% CI 1.07–2.86) were associated with significantly increased risk of treatment failure.

**Conclusions:**

Home visits, compared with out-patient clinic care, significantly reduce treatment failure in individuals with schizophrenia undergoing LAI treatment.

Schizophrenia is a chronic and debilitating mental disorder characterised by diverse impairments in cognition, mood and reality testing.^1^ Approximately half of patients fail to adhere to treatment plans, leading to relapse.^2,3^ Because relapses can result in progressive worsening of clinical symptoms, self-harm, reduced social functioning and increased healthcare costs,^4^ prevention of relapse is crucial.

Long-acting injectable antipsychotic medications (LAIs) are known to enhance adherence by reducing the frequency of administration, ensuring consistent dosing and providing stable pharmacokinetics, while also enabling regular monitoring of treatment.^5^ Therefore, it has been demonstrated that LAIs are superior to oral medications in prevention of relapse.^6^ Despite their proven efficacy, some people are unable to benefit from LAIs due to barriers to attending out-patient clinics. Indeed, it has been reported that 28.8% of individuals discontinue LAIs within 1 year of initiation.^7^

There are several reasons why schizophrenia subjects may be unable to attend out-patient clinics. These include a lack of insight into their illness, leading to low treatment motivation;^8^ exacerbation of delusions or anxiety triggered by crowded public spaces;^9^ avoidance of social contact due to negative symptoms;^10^ and cognitive impairments that make regular attendance challenging.^11^ As a result, maintaining consistent LAI administration for those unable to visit out-patient clinics remains a significant challenge.

Home visits have been implemented in many countries, each demonstrating unique characteristics and effectiveness.^12–14^ In Japan, home visit systems allow physicians to visit the patient’s home weekly to monthly to provide services such as symptom evaluation, medication management and psychoeducation.^15^ This system enables LAI administration at the patient’s home for those unable to attend out-patient clinics. However, few studies have evaluated the effectiveness of home visits in prevention of treatment failure by direct administration of LAIs in the patient’s home.^16^

This study utilised electronic medical records from Seijin Hospital to conduct a retrospective cohort analysis. Schizophrenia individuals who had been treated with LAIs during hospitalisation and subsequently discharged were included in this study. The outcome was compared between the two groups: those receiving home visits and those attending out-patient clinics. The aim of this study was to clarify whether the administration of LAIs through home visits reduces treatment failure compared with out-patient care, thereby evaluating the utility of home visits for individuals treated with LAIs.

## Method

### Subjects

We conducted a retrospective cohort study using the electronic medical records of Seijin Hospital. Seijin Hospital is located in the north-eastern Tokyo Metropolitan Area, which includes Adachi, Arakawa and Katsushika wards. This urban zone spans approximately 98.21 km^2^ and had a population of 1 365 611 as of 2020, with a population density of 13 905 persons/km^2^. The elderly population (aged ≥65 years) accounted for 25.0%, slightly below the national average. Socioeconomically, the area contains wards with relatively low income levels compared with other parts of Tokyo. For example, Adachi ward had the lowest average per capita income among Tokyo’s 23 special wards in 2023, at approximately 3.55 million yen.^17,18^ The inclusion criteria were individuals diagnosed with schizophrenia based on DSM-5^19^ and who were newly admitted to the psychiatric emergency ward. There were no age or gender restrictions. Among these subjects, we included those who received LAIs during their hospitalisation and were discharged between 1 April 2020 and 31 March 2023. Although this study targeted patients treated with LAIs during hospitalisation, some had already been prescribed LAIs prior to admission and continued treatment during their hospital stay.

Data were extracted from the electronic medical records of Seijin Hospital. Information collected included age, gender, voluntary or involuntary admission status, DSM-5 diagnosis, duration of illness, history of psychiatric hospitalisations, use of physical restraint during hospitalisation, types of oral medication prescribed, Global Assessment of Functioning (GAF) scores, history of modified electroconvulsive therapy (mECT) during hospitalisation, follow-up methods following discharge and whether the subject lived alone following discharge. These variables were selected based on predictors of rehospitalisation reported in previous studies.^20,21^ Additionally, the number of days until psychiatric rehospitalisation, discontinuation of out-patient visits or death was recorded.

Subjects were categorised into two groups based on their follow-up care following discharge, as determined by their attending physician: the out-patient care and home visit groups. Observations began at the time of discharge and ended 1 year later. Those who were referred at discharge to institutions outside the hospital’s follow-up system, and whose follow-up data were consequently unavailable in our electronic medical record system, were excluded. These excluded subjects had been referred to psychiatric out-patient clinics – either those located closer to their home or to their previous out-patient psychiatrists prior to admission. This referral pattern reflects a unique feature of the Japanese healthcare system, whereby patients are not restricted by geographic or administrative boundaries when selecting medical institutions. As a result, such planned transitions are a common and appropriate aspect of psychiatric care in Japan.

Although it is possible that the administration of LAIs could be compelled as part of a treatment plan under the Medical Treatment and Supervision Act at specific medical institutions, none of the patients in the present study were undergoing treatment under this act. Therefore, all patients in this study initiated and continued LAI treatment based on consent, and there were no cases of compulsory administration.

Regarding the LAI administration process, there were differences between the two groups. In the home visit group, the attending physician (psychiatrist) both prescribed the LAI and administered the injection during each home visit, ensuring consistency of the clinician directly involved in the injection process. In contrast, for those in the out-patient care group, the LAI was prescribed by their attending physician but the injection was administered by the nursing staff on duty in the out-patient clinic at the time of the appointment. In the out-patient care group, the LAI was administered at the out-patient clinic located within Seijin Hospital, a specialised psychiatric hospital.

The primary outcome was the risk of treatment failure, defined as either psychiatric rehospitalisation, discontinuation of treatment or death.

### Statistical analysis

Statistical analyses were performed to compare the time to treatment failure between the two groups using the Kaplan–Meier method. Statistical significance was assessed using univariate analysis with the log-rank test, and multivariate analysis with a Cox proportional hazards model. Statistical analyses were conducted using JMP version 18.1.0 (SAS Institute Inc., Cary, NC, USA).

This study was approved by the Ethics Committee of Seijin Hospital (approval no. 2403). Because this was a retrospective, non-interventional study, informed consent was not required. Rather, information about the study was made available on the Seijin Hospital website, allowing subjects to opt out. All data were fully anonymised and managed to ensure that individuals could not be identified.

## Results

A total of 304 patient records were extracted from the electronic medical records of Seijin Hospital. These patients had been diagnosed with schizophrenia, admitted to the hospital, treated with LAIs during hospitalisation and subsequently discharged between 1 April 2020 and 31 March 2023. No patients opted out of the study. Sixty-two patients who were referred to other institutions at discharge and whose follow-up data were unavailable were excluded, leaving 242 patients for analysis. At the time of discharge, patients were categorised by their attending physicians into either the home visit group (125 patients) or the out-patient care group (117 patients). Table 1 provides the baseline characteristics of both groups.

**Table 1:** Comparison of basic characteristics between home visit and out-patient groups

Characteristic	Home visit (*n* = 125), *n* (%)	Out-patient (*n* = 117), *n* (%)	*P*-value
Age (years)	53 ± 20	43 ± 21	<0.001^a^**
Male	46 (36.8)	51 (43.6)	0.281^b^
LAI			0.004^a^**
Paliperidone	96 (76.8)	63 (53.8)	
Aripiprazole	26 (20.8)	44 (37.6)	
Risperidone	2 (1.6)	5 (4.2)	
Haloperidol	1 (0.8)	3 (2.6)	
Fluphenazine	0 (0)	2 (1.7)	
Initiated LAI during index hospitalisation	96 (76.8)	78 (66.7)	0.108^b^
GAF	62 ± 15	62 ± 17	0.800^a^
Time since disease onset (years)	15 ± 25	10 ± 12	0.001^a^**
Involuntary admission	111 (88.8)	91 (77.8)	0.021^b^*
Number of previous psychiatric hospitalisations			0.090^a^
0	27 (23.1)	25 (20)	
1	19 (16.2)	35 (28)	
2 or more	71 (60.7)	65 (52)	
Duration of hospital stay (days)	36 ± 24	32 ± 27	0.268^a^
Seclusion and restraint during hospitalisation	87 (69.6)	67 (57.2)	0.046^b^*
ECT during hospitalisation	37 (29.6)	30 (25.6)	0.492^b^
Antipsychotic polytherapy at discharge	70 (56.0)	62 (53.0)	0.639^b^
Concomitant use of (non-)benzodiazepines at discharge	71 (56.8)	60 (51.3)	0.389^b^
Living alone following discharge	35 (28)	17 (14.5)	0.011^b^*

ECT, electroconvulsive therapy; GAF, Global Assessment of Functioning; LAI, long-acting injectable antipsychotic medication.

Age, GAF, time since disease onset and duration of hospital stay are presented as median ± interquartile range.

a Mann–Whitney *U*-test.

b Chi-square test.

*P < 0.05, **P < 0.01.

There were no significant differences between the two groups in terms of gender, GAF score, initiation of LAI during index hospitalisation, history of psychiatric hospitalisations, length of hospitalisation, use of mECT during hospitalisation, concomitant use of two or more antipsychotics at discharge or concomitant use of benzodiazepines. However, patients in the home visit group were significantly older, had a longer duration of illness, were more likely to have been involuntarily admitted, experienced more frequent use of physical restraint during hospitalisation and were more likely to live alone following discharge. Additionally, there were significant differences between groups in the types of LAI used.

**Fig. 1:**
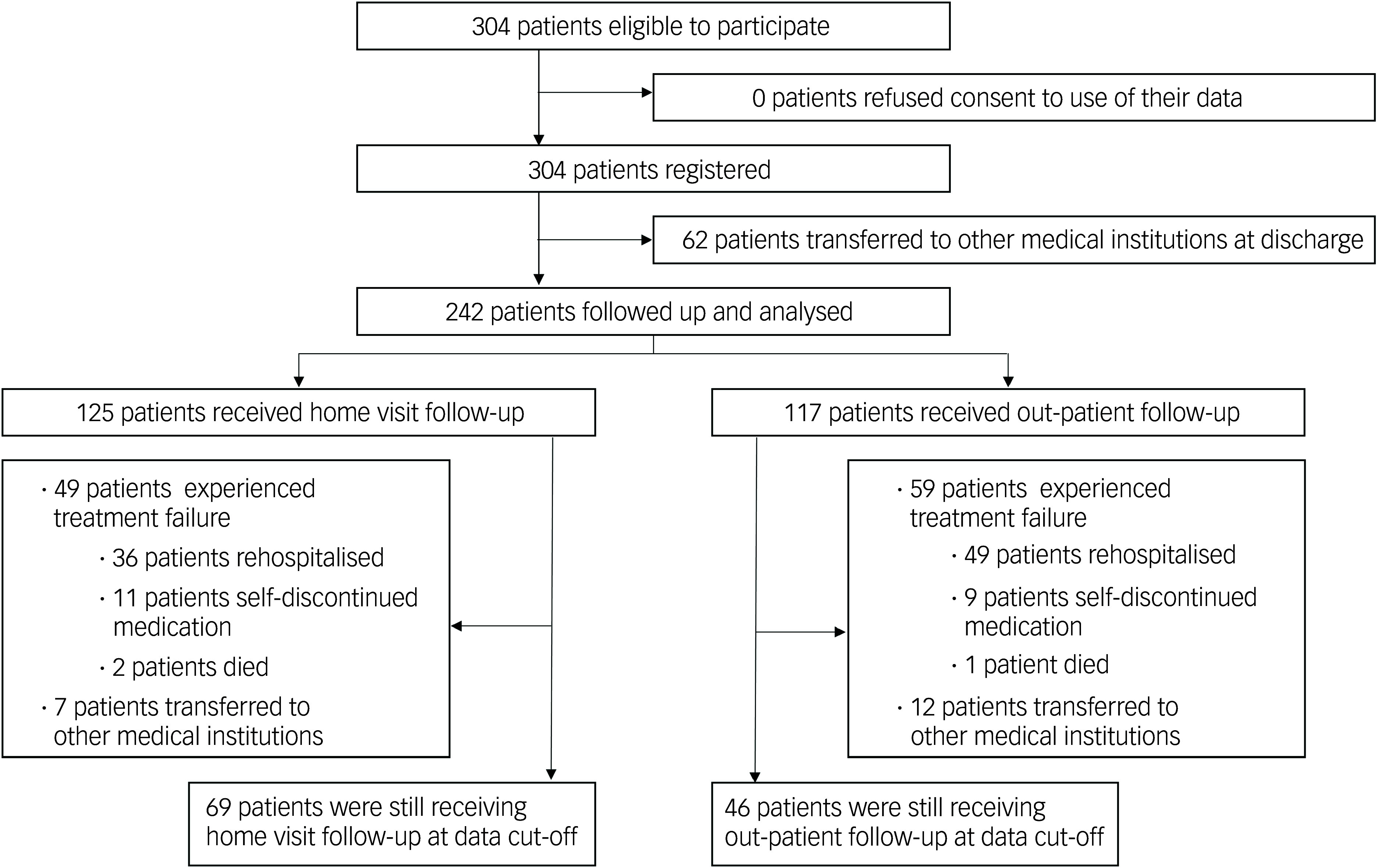
Flow diagram of the study. During the enrolment period, 125 subjects received home visits while 117 received out-patient clinic treatment, and all were followed up for 1 year. Treatment failure was defined as either psychiatric rehospitalisation, self-discontinuation of treatment or death. In the home visits group, 49 cases met the criteria for treatment failure, while in the out-patient group 58 cases met the criteria.

Both groups were followed for 1 year. In the home visit group, 36 patients were rehospitalised, 11 discontinued treatment and 2 died, resulting in a total of 49 treatment failures. Seven patients were referred to other institutions with their physician’s consent. After 1 year, 69 patients continued treatment. In the out-patient care group, 49 patients were rehospitalised, 9 discontinued treatment and 1 died, for a total of 59 treatment failures. Twelve patients were referred to other institutions with their physician’s consent. After 1 year, 46 patients continued treatment (Fig. 1). These transfers were primarily due to patient preferences or logistical issues such as relocation, and were carried out with mutual agreement between the patient and their physician. In the Japanese healthcare system, patients have the freedom to choose or change their treating institution at any time, irrespective of their clinical status. Importantly, these cases do not reflect clinical deterioration requiring more specialised care, nor do they represent unilateral discontinuation by the patient. Therefore, these transitions were regarded as planned and analysed separately from treatment failure. Among the three deaths, one individual in the out-patient group and one in the home visit group died by suicide. The other death in the home visit group was from an undetermined cause.

Table 2 summarises the 1-year clinical end-points for both groups. These include treatment failure, rehospitalisation, treatment discontinuation, death, transfer to another institution, continued treatment and adherence rate to LAI treatment at the end of the observation period. As a result, it was shown that the home visit group had a significantly lower rehospitalisation rate and a significantly higher continued treatment rate. Regarding LAI adherence at the end of observation, no significant difference was observed between the groups.

**Table 2:** One-year outcomes by group (home visit versus out-patient care)

Outcome	Home visit (*n* = 125), *n* (%)	Out-patient (*n* = 117), *n* (%)	*P*-value
Treatment failure	49 (39.2)	59 (50.4)	0.103^a^
LAI adherence at end	41/49 (83.7)	52/59 (88)	0.698^a^
Rehospitalisation	36 (28.8)	49 (41.9)	0.046^a^*
Treatment discontinuation	11 (8.8)	9 (7.7)	0.937^a^
Death	2 (1.6)	1 (0.9)	1.000^b^
Planned transfer	7 (5.6)	12 (10.3)	0.268^a^
LAI adherence at end	5/7 (71.4)	12/12 (100)	0.123^b^
Continued treatment	69 (55.2)	46 (39.3)	0.019^a^*
LAI adherence at end	56/69 (81.1)	42/46 (91.3)	0.217^a^

LAI, long-acting injectable antipsychotic medication.

a Chi-square test.

b Fisher’s exact test.

*P < 0.05.

### Kaplan–Meier analysis

**Fig. 2:**
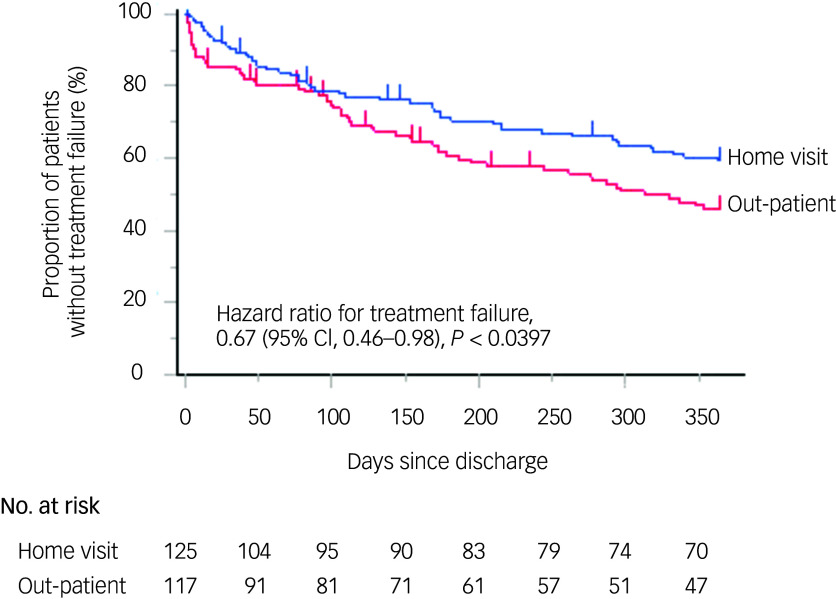
Kaplan–Meier curve of treatment failure in home visits versus out-patient groups. The curve compares treatment failure rates (rehospitalisation, discontinuation or death) between home visits (blue) and out-patient (red) groups following discharge. The hazard ratio for treatment failure in the home visits group was 0.67 (95% CI, 0.46–0.98, *P* < 0.05), showing a significantly lower risk than the out-patient group. The number of patients at risk over time is shown below the *x* axis.

Kaplan–Meier survival curves illustrating treatment failure over the 1-year follow-up period are shown in Fig. 2. Univariate analysis revealed that the hazard ratio for treatment failure in the home visit group was significantly lower than that in the out-patient care group (0.67, 95% CI 0.46–0.98, *P* < 0.0397).

### Cox proportional hazards model

As shown in Table 1, several baseline characteristics differed significantly between the two groups; therefore, multivariate analysis using the Cox proportional hazards model was performed to adjust for these variables (Table 3). The results indicated that follow-up care via home visits was associated with a significantly lower risk of treatment failure (hazard ratio 0.62, 95% CI 0.40–0.97, *P* = 0.035). In contrast, a history of two or more psychiatric hospitalisations (hazard ratio 2.32, 95% CI 1.28–4.37, *P* = 0.007) and living alone following discharge (hazard ratio 1.77, 95% CI 1.07–2.86, *P* = 0.022) were significantly associated with an increased risk of treatment failure. No other covariates showed significant hazard ratios. These trends remained consistent across models adjusted for different covariates.

**Table 3:** Hazard ratios for outcome of treatment failure by multivariate analysis using Cox proportional hazards model

Outcome	Hazard ratio	95% CI	*P*-value
Home visit	0.622	0.40–0.97	0.035*
Age (years)	1.004	0.98–1.02	0.648
Gender	0.96	0.63–1.47	0.841
LAI			0.471
Paliperidone	1.00		
Aripiprazole	1.24	0.80–1.92	
Risperidone	1.53	0.54–4.36	
Haloperidol	0.32	0.04–2.36	
Fluphenazine	2.07	0.45–9.47	
GAF	0.99	0.98–1.01	0.268
Time since disease onset (years)	0.997	0.97–1.02	0.804
Involuntary admission	0.86	0.45–1.64	0.646
Number of previous psychiatric hospitalisations, ≥2	2.32	1.28–4.37	0.007**
Duration of hospital stay (days)	1.00	0.98–1.02	0.612
Seclusion and restraint during hospitalisation	1.09	0.67–1.84	0.725
ECT during hospitalisation	1.45	0.94–2.22	0.088
Antipsychotic polytherapy at discharge	1.34	0.88–2.06	0.173
Concomitant use of (non-)benzodiazepines	0.78	0.52–1.18	0.246
Living alone following discharge	1.77	1.07–2.86	0.022*

ECT, electroconvulsive therapy; GAF, Global Assessment of Functioning; LAI, long-acting injectable antipsychotic medication.

*P* < 0.05, **P < 0.01.

## Discussion

The aim of this study was to clarify whether follow-up care via home visits reduces treatment failure in schizophrenia subjects treated with LAIs. In the adjusted model, follow-up care through home visits was significantly associated with a reduced risk of treatment failure (hazard ratio 0.62, 95% CI 0.40–0.97) compared with out-patient clinic. Conversely, a history of two or more psychiatric hospitalisations (hazard ratio 2.32, 95% CI 1.28–4.37) and living alone following discharge (hazard ratio 1.77, 95% CI 1.07–2.86) were significantly associated with an increased risk of treatment failure. These findings suggest that home visit follow-up care is effective in preventing treatment failure in schizophrenia individuals treated with LAIs. Additionally, living alone following discharge and a history of multiple psychiatric hospitalisations were significantly associated with an increased risk of treatment failure, findings that are consistent with previous studies.^22,23^ These factors highlight the importance of providing targeted support to vulnerable patients, particularly those who live alone or have a history of frequent hospitalisations.

The lower risk of treatment failure in the home visit group may be attributable to several interacting factors. First, compared with out-patient care, which requires ongoing appointments and clinic visits, LAI administration during home visits may ensure adherence regardless of the patient’s motivation or level of functioning. In Table 2, LAI adherence in the home visit group shows a tendency to be poorer compared with the out-patient group, although this difference is not statistically significant. In the out-patient group, some people who were hesitant to receive LAI injections may have discontinued clinic visits without explicitly refusing treatment. In such cases, LAI administration may appear to have continued until formal discontinuation, resulting in an apparently high adherence rate despite poor engagement. This might partly explain why LAI adherence rates appear similar between groups, despite different rates of treatment failure. Second, the difference in the LAI administration process might have influenced the results. The consistency of having the same physician administer LAI during home visits could have fostered a stronger therapeutic alliance compared with the out-patient setting where the administering nurse varied, potentially contributing to the lower rate of treatment failure observed in the home visit group. Third, observing patients in their home environment allows clinicians to gain deeper insight into their daily functioning and detect early warning signs of relapse, enabling more timely and targeted interventions. Fourth, the structure of the Japanese healthcare system may also contribute to this difference. Whereas out-patient treatment can be passively discontinued by simply not attending appointments, home visit services generally require patients to actively communicate their intention to stop care, which may raise the psychological threshold for treatment discontinuation. These mechanisms are considered to have acted synergistically to reduce treatment failure, and provide direction for optimising future community-based care strategies.

It should be noted that, in this study, a significant difference was observed in the types of LAI used between the home visit and out-patient groups, with a particularly noticeable difference in the prescription rates of aripiprazole LAI and paliperidone LAI (Table 1). Although it is conceivable that this disparity in LAI type could have influenced the results, in the multivariate Cox proportional hazards model analysis that we conducted the influence of LAI type was adjusted for and, even following this adjustment, home visits remained significantly associated with a reduced risk of treatment failure (Table 3). Furthermore, a large-scale network meta-analysis by Ostuzzi et al^24^ reported no significant difference in overall efficacy between aripiprazole LAI and paliperidone LAI in preventing relapse among individuals with schizophrenia. Therefore, it is considered unlikely that the superiority of home visits observed in this study can be primarily explained by differences in the types of LAI prescribed.

From the perspective of preventing treatment failure, incorporating home visit follow-up care alongside LAIs, whenever feasible, could provide substantial benefits. However, the feasibility of home visits varies significantly depending on the country and its healthcare system. In Europe, differences in accessibility to home visits exist not only between countries but also within regions.^25^ In countries such as Bangladesh, Ethiopia and India, government-led, large-scale home visit programmes have been implemented, but challenges such as low coverage remain prevalent.^26^

In contrast, Japan, with one of the most rapidly ageing populations globally,^27^ has been promoting home medical care in general, which is not limited to psychiatry, through policies supporting home visits.^28^ This community comprehensive care system, started in 2017, is officially abbreviated as ‘also comprehensive care’, implying that this system is for comprehensive care in general, but it ‘also’ addresses mental disorders. The relatively high reimbursement rates (8880 yen per patient) for home visits in the Japanese healthcare system have facilitated widespread implementation of such follow-up care. Nevertheless, establishing similar systems globally may be challenging due to differences in healthcare infrastructure and financial resources.

This study has several limitations. First, this study was based on data from a single medical institution, which may limit the generalisability of the findings to broader patient populations. Second, the retrospective cohort design and the non-randomised assignment of patients to either home visit or out-patient clinic, determined by the attending physician, limit the ability to draw causal inferences. Indeed, there were significant differences in baseline characteristics between the two groups. However, because most of these biases seen in home visits groups – longer duration of illness, involuntary admission, seclusion and restraint during hospitalisation and living alone after discharge – were towards the worse outcome, it is unlikely that the present findings are mere artefact due to selection bias.

In conclusion, this study investigated whether the introduction of home visits for schizophrenia subjects treated with LAIs is effective in preventing treatment failure. The results demonstrated that home visits significantly reduce the risk of treatment failure. To enhance cost-effectiveness by reducing symptom exacerbations and rehospitalisations, actively implementing home visits for those in need of such support is recommended.

Further research is needed to elucidate the mechanisms by which home-based LAI administration reduces treatment failure, thereby enhancing its therapeutic effectiveness. Moreover, comprehensive investigations into the cost-effectiveness and optimal implementation strategies of home-based LAI administration are warranted, to maximise the therapeutic potential of LAIs and improve outcomes for individuals with schizophrenia.

## Data Availability

Due to the nature of this research, participants in this study did not agree to their data being shared publicly.

## References

[1] van Os J , Kapur K . Schizophrenia. Lancet 2009; 374: 63545. 19700006 10.1016/S0140-6736(09)60995-8

[2] Haddad PM , Brain C , Scott J. Nonadherence with antipsychotic medication in schizophrenia: challenges and management strategies. Patient Relat Outcome Meas 2014; 5: 43–62. 25061342 10.2147/PROM.S42735 PMC4085309

[3] Sendt KV , Tracy DK , Bhattacharyya S. A systematic review of factors influencing adherence to antipsychotic medication in schizophrenia-spectrum disorders. Psychiatry Res 2015; 225: 14–30. 25466227 10.1016/j.psychres.2014.11.002

[4] Thornicroft G , Brohan E , Rose D , Sartorius N , Leese M. Global pattern of experienced and anticipated discrimination against people with schizophrenia: a cross-sectional survey. Lancet 2009; 373: 408–15. 19162314 10.1016/S0140-6736(08)61817-6

[5] Park EJ , Amatya S , Kim MS , Park JH , Seol E , Lee H , et al. Long-acting injectable formulations of antipsychotic drugs for the treatment of schizophrenia. Arch Pharm Res 2013; 36: 651–9. 23543652 10.1007/s12272-013-0105-7

[6] Kishimoto T , Hagi K , Kurokawa S , Kane JM , Correll CU. Long-acting injectable versus oral antipsychotics for the maintenance treatment of schizophrenia: a systematic review and comparative meta-analysis of randomised, cohort, and pre-post studies. Lancet Psychiatry 2021; 8: 387–404. 33862018 10.1016/S2215-0366(21)00039-0

[7] Auxilia AM , Buoli M , Caldiroli A , Carnevali GS , Tringali A , Nava R , et al. High rate of discontinuation during long-acting injectable antipsychotic treatment in patients with psychotic disorders. Biomedicines 2023; 11: 314. 36830850 10.3390/biomedicines11020314 PMC9953565

[8] Lysaker PH , Buck KD , Salvatore G , Popolo R , Dimaggio G. Lack of awareness of illness in schizophrenia: conceptualizations, correlates and treatment approaches. Expert Rev Neurother 2009; 9: 1035–43. 19589052 10.1586/ern.09.55

[9] Freeman D , Emsley R , Dunn G , Fowler D , Bebbington P , Kuipers E , et al. The stress of the street for patients with persecutory delusions: a test of the symptomatic and psychological effects of going outside into a busy urban area. Schizophr Bull 2015; 41: 971–9. 25528759 10.1093/schbul/sbu173 PMC4466180

[10] Worswick E , Dimic S , Wildgrube C , Priebe S. Negative symptoms and avoidance of social interaction: a study of non-verbal behaviour. Psychopathology 2018; 51: 1–9. 29224023 10.1159/000484414

[11] Zaragoza Domingo S , Bobes J , García-Portilla MP , Morralla C. Cognitive performance associated to functional outcomes in stable outpatients with schizophrenia. Schizophr Res Cogn 2015; 2: 146–58. 29379764 10.1016/j.scog.2015.03.002 PMC5779297

[12] Okie S. Home delivery—bringing primary care to the housebound elderly. N Engl J Med 2008; 359: 2409–12. 19052122 10.1056/NEJMp0808067

[13] Stall N , Nowaczynski M , Sinha SK. Back to the future: home-based primary care for older homebound Canadians: part 1: where we are now. Can Fam Physician 2013; 59: 237–40. 23486788 PMC3596195

[14] Boerma WGW , Groenewegen PP. GP home visiting in 18 European countries adding the role of health system features. Eur J Gen Pract 2001; 7: 132–7.

[15] Yokobayashi K , Matsushima M , Watanabe T , Fujinuma Y , Tazuma S. Prospective cohort study of fever incidence and risk in elderly persons living at home. BMJ Open 2014; 4: e004998. 10.1136/bmjopen-2014-004998 PMC4091458 25009132

[16] Chang HH , Vaughn LM , Liu D. Rural ambulatory care pharmacists providing in-clinic and home visit services improve adherence to long-acting injectable antipsychotics. Ment Health Clin 2024; 14: 229–32. 38835813 10.9740/mhc.2024.06.229 PMC11147658

[17] Ministry of Internal Affairs and Communications. Survey of Municipal Tax Assessment Status for Fiscal Year 2023. Ministry of Internal Affairs and Communications, 2024 (https://www.soumu.go.jp/main_sosiki/jichi_zeisei/czaisei/czaisei_seido/ichiran09_23.html).

[18] Japan Medical Analysis Platform (JMAP). *Tokyo Northeastern Medical District*. JMAP, 2025 (https://jmap.jp/cities/detail/medical_area/1306).

[19] American Psychiatric Association (APA). Diagnostic and Statistical Manual of Mental Disorders: DSM-5 5th ed. APA, 2013.

[20] Sfetcu R , Musat S , Haaramo P , Ciutan M , Scintee G , Vladescu C , et al. Overview of post-discharge predictors for psychiatric re-hospitalisations: a systematic review of the literature. BMC Psychiatry 2017; 17: 227. 28646857 10.1186/s12888-017-1386-z PMC5483311

[21] Hatta K , Katayama S , Ishizuka T , Sudo Y , Nakamura M , Hasegawa H , et al. Real-world effectiveness of antipsychotic treatments in 1011 acutely hospitalized patients with schizophrenia: a one-year follow-up study. Asian J Psychiatry 2022; 67: 102917. 10.1016/j.ajp.2021.102917 34875558

[22] Dean C , Gadd EM. Home treatment for acute psychiatric illness. BMJ 1990; 301: 1021–3. 2249049 10.1136/bmj.301.6759.1021 PMC1664005

[23] Olfson M , Mechanic D , Hansell S , Boyer CA , Walkup J , Weiden PJ. Predicting medication noncompliance after hospital discharge among patients with schizophrenia. Psychiatr Serv 2000; 51: 216–22. 10655006 10.1176/appi.ps.51.2.216

[24] Ostuzzi G , Bertolini F , Del Giovane C , Tedeschi F , Bovo C , Gastaldon C , et al. Maintenance treatment with long-acting injectable antipsychotics for people with nonaffective psychoses: a network meta-analysis. Am J Psychiatry 2021; 178: 424–36. 33596679 10.1176/appi.ajp.2020.20071120

[25] Genet N , Boerma WG , Kringos DS , Bouman A , Francke AL , Fagerström C , et al. Home care in Europe: a systematic literature review. BMC Health Serv Res 2011; 11: 207. 21878111 10.1186/1472-6963-11-207 PMC3170599

[26] McPherson R , Hodgins S. Postnatal home visitation: lessons from country programs operating at scale. J Glob Health 2018; 8: 010422. 29977530 10.7189/jogh.08.010422 PMC6005634

[27] Tamiya N , Noguchi H , Nishi A , Reich MR , Ikegami N , Hashimoto H , et al. Population ageing and wellbeing: lessons from Japan’s long-term care insurance policy. Lancet 2011; 378: 1183–92. 21885099 10.1016/S0140-6736(11)61176-8

[28] Ministry of Health, Labour and Welfare (MHLW). Promotion of Long-term Care and Home Care. MHLW, 2012 (https://www.mhlw.go.jp/seisakunitsuite/bunya/kenkou_iryou/iryou/zaitaku/dl/zaitakuiryou_all.pdf).

